# Dual actions on gout flare and acute kidney injury along with enhanced renal transporter activities by Yokuininto, a Kampo medicine

**DOI:** 10.1186/s12906-019-2469-9

**Published:** 2019-03-12

**Authors:** Seung Hoon Lee, Ho-Sung Lee, Gunhyuk Park, Sung-Man Oh, Dal-Seok Oh

**Affiliations:** The K-herb Research Center, Korea Institute of Oriental Medicine, 1672 Yuseong-daero, Yuseong-gu, Daejeon, 34054 Republic of Korea

**Keywords:** SLC22A8, SLC2A9, Kidney, Yokuininto

## Abstract

**Background:**

Prolonged hyperuricemia is associated with kidney disease or gouty arthritis. Whether Yokuininto, a commercially available Kampo medicine that has been used for osteoarthritis or rheumatoid arthritis, can exhibit anti-hyperuricemic and inflammatory effects remains elusive. In the present study, Yokuininto exerts multiple homeostatic action on serum uric acid (sUA) levels by blocking pro-inflammatory cytokine activities and inducing uricosuric function with anti-renal injury functions.

**Methods:**

The sUA was measured in potassium oxonate (PO)-administered mice. Renal transporter uptake assays were performed using HEK293 cells overexpressing OAT1, OCT2 or OAT3, MDCKII cells overexpressing BCRP, and Xenopus oocytes overexpressing OAT3 or URAT1. Immunoblot and ELISA assays were performed to detect the molecules (OAT3, GLUT9, XO, NGAL, KIM-1 and IL-1α) in various human kidney cell lines. Cell viability analysis was performed to evaluate the cytotoxicity of Yokuininto [Ephedrine + pseudoephedrine 21.94%; Paeoniflorin 35.40% and Liquiritin 16.21% relatively measured by the ratios (HR-MS2 intensity / HR-MS1 intensity)].

**Results:**

Yokuininto (300 mg/kg) significantly reduced sUA by approximately 44% compared to that of PO-induced mice. The OAT3 levels were decreased in PO-induced hyperuricemic condition, whereas the GLUT9 transporter levels were markedly increased. However, PO did not alter the levels of URAT1. Yokuininto significantly inhibited the lipopolysaccharide (LPS)-induced secretion of IL-1α by approximately 63.2% compared to the LPS-treated macrophages. In addition, Yokuininto inhibited nitric oxide synthesis by approximately 33.7 (500 µg/mL) and 64.6% (1000 µg/mL), compared to that of LPS-treated macrophages. Yokuininto markedly increased xanthine oxidase inhibition activity. Furthermore, interleukin-1α (IL-1α), a pro-inflammatory cytokine, elevated neutrophil gelatinase-associated lipocalin (NGAL) and kidney injury molecule-1 (KIM-1) activities in LLC-PK1 cells. Expression of renal inflammatory biomarkers, NGAL and KIM-1, was reduced under the Yokuininto treatment by 36.9 and 72.1%, respectively.

**Conclusions:**

Those results suggest that Yokuininto may suppress inflammation and protect against kidney dysfunction in hyperuricemia. The present findings demonstrated that Yokuininto lowered sUA through both increased uric acid excretion and decreased uric acid production. Our results may provide a basis for the protection of prolonged hyperuricemia-associated kidney injury with uric acid-lowering agents such as Yokuininto.

**Electronic supplementary material:**

The online version of this article (10.1186/s12906-019-2469-9) contains supplementary material, which is available to authorized users.

## Background

Prolonged systemic hyperuricemia has been regarded as an etiology of gout and it also causes inflammation and uric acid congestion in kidney cortex. Increased tubular reabsorption or the reduced tubular secretion in the basolateral side of the proximal tubule membrane occurs in the uric acid-affected glomeruli [1–3]. Previous studies have reported that two basolateral membrane transporters, the organic anion transporter 3 (OAT3), which possesses secretory functions, and the glucose transporter 9 (GLUT9), which performs influx functions, are closely associated with the pathophysiology of hyperuricemia [4].

Interleukin-1 alpha (IL-1a) is a powerful inflammatory cytokine that regulates both adaptive and innate immunity. As such, it is implicated in the development of multiple autoimmune and inflammatory diseases such as arthritis. IL-1α is potent inflammatory cytokine that activates the inflammatory process, and the deregulated signaling causes devastating diseases manifested by acute or chronic inflammation. IL-1α binds to the receptor and then, it has alike pro-inflammatory functions [5, 6]. IL-1α can be found asa cell-bound molecule on the plasma membrane in epithelial cells  and can be considered as tissue damage and then, begins triggering the early phases of gout flare with severe pains [7].

Acute kidney injury is one of the major kidney disease characterized by rapid and excessive loss of the renal function, which leads to the aberrant accumulation of nitrogenous metabolic wastes (e.g. urea and creatinine) and imbalance of water, electrolytes and acid-base reactions [8, 9]. Recently, neutrophil gelatinase-associated lipocalin (NGAL) and kidney injury molecule-1 (KIM-1) have been rigorously tested as robust biomarkers for acute kidney injury. The use of several antioxidants corroborated previous findings regarding the avoidance of gout flare with IL-1 blockers. NGAL is expressed by IL-1 in epithelial cells during inflammation [10]. KIM-1 is significantly upregulated in the kidney after injury and related to inflammation such as chronic kidney dysfunction (CKD) and polycystic kidney disease (PKD) [11, 12].

To test the hypothesis that Yokuininto, a Kampo medicine that has been used for osteoarthritis [13] or rheumatoid arthritis [14], can be applied to treat hyperuricemia and gouty arthritis, we investigated the uric acid modulating effects of Yokuininto on serum level of uric acid, XO inhibiting activity, and OAT3 and GLUT9 transporter activities. In addition, its anti-inflammatory effect was assessed by monitoring the activities of acute kidney injury biomarkers, NGAL and KIM-1, which are known to mediate the inflammatory response upon the cytokine IL-1α stimulation in macrophages.

## Methods

### Materials and reagents

Yokuininto (K-25) Kampo medicine was donated from Kracie (Seoul, Korea). The dried extract powder of K-25 (Lot No. 15071713, Kracie) was used in this present study. The components and indications of K-25, a Kampo medicine, are described in details [13, 15, 16]. Stock solution was diluted in DMSO at 50 mg/mL and stored at − 20 °C. IL-1α recombinant was purchased from Thermo scientific (Rockford, IL, USA). Allopurinol (A8003), LPS (L6529) and potassium oxonate (PO, 156124) were purchased from Sigma-Aldrich (St. Louis, MO, USA).

### Cell culture

We purchased four cell lines from American Type Culture Collection cell line (ATCC, MD, USA) and their ATCC® Numbers were Raw 264.7 (TIB-71™), LLC-PK1 (CL-101™), HEK293 (CCL-1573™) and MDCK (CCL-34™). All the four cell lines, i.e., Raw 264.7, LLC-PK1, HEK293, and MDCK cells, were kept in DMEM (Gibco, MD, USA) supplemented with 10% FBS, 1% penicillin/streptomycin and cultured in a humidified incubator at 37 °C in 5% CO_2_.

### Measurement of cytotoxicity

Raw 264.7 and LLC-PK1 cells were seeded into 96-well plates and then the cells were treated with K-25 in media for 24 h. Cell viabilities were determined using the Ez-cytox assay kit (Dogen, Republic of Korea) according to the manufacturer’s instructions.

### Immunoblot analysis

The protein samples were prepared in RIPA lysis buffer (89,900, Thermo Fisher Scientific). Samples were separated on 12% SDS-polyacrylamide gels and then, transferred to polyvinylidenefluoride (PVDF) membranes, which were blocked with 5% dried milk in PBS containing 0.5% Tween-20. The blots were incubated with the appropriate antibodies at a dilution of 1:1000. The antibodies were those against OAT1 (TA322017, OriGene), OAT3 (TA321636, OriGene), ABCG2 (sc-58,222, Santa cruz), URAT1 (LS-C335533, LsBio), GLUT9 (NBP1–06271, novusbio), KIM-1 (ab47635, abcam), NGAL (ab63929, abcam) enzymes. Images of the blotted membranes were obtained using a LAS-4000 lumino-image analyzer (GE Healthcare Life Sciences, Republic of Korea).

### Cytokine antibody array

At the end of each treatment, the culture medium was collected in eppendorf tubes. After centrifugation at 10,000 × g for 10 min, the supernatants were assayed for secreted mediators using RayBio C-Series Mouse Cytokine Antibody Array C2000 (AAM-CYT-2000-8, RayBiotech) according to the manufacturer’s instructions.

### Measurement of nitric oxide

The nitrite in culture supernatants was measured as an indicator of nitric oxide (NO) production. An aliquot of the culture supernatant was mixed with a volume of Griess reagent (G2930, Promega), and the absorbance at 570 nm was determined using a microplate reader.

### NGAL, KIM-1 and transporters measurements

NGAL and KIM-1 were determined by an ELISA assay kit (Cloud-clone, USA) according to the manufacturer’s instructions. LLC-PK1 cells (1 × 10^6^ cells/6 well) were treated with various concentrations of IL-1α (0–100 ng/ml) for 24 h. After incubation, the medium was collected, and centrifuged at 3000 rpm. The supernatants were carefully transferred into fresh tubes. Supernatants (50 μl) were added to 50 μl of a 1/1 mixture of 0. 01 mol/L PBS (pH 7.0). Plates were incubated at 4 °C for overnight. Transporters were measured using an ELISA kit (Mybiosource, USA) according to the manufacturer’s recommendation.

### Transporter uptake assays

Transporter uptake assays were performed as described previously [17]. In brief, the transporter expressing HEK293 cells were seeded on BD poly-D-lysine 24 well microplates with a density of approximately 1 × 10^5^ cells/wells in DMEM supplemented with 10% FBS. Uptake of [^3^H]para-aminohippuric acid (PAH) for OAT1, [^3^H]estrone sulfate (ES) for OAT3 and [^3^H]1-methyl-4-phenylpyridinium (MPP^+^) for OCT2 were assayed at 37 °C in Ringer’s solution (130 mM NaCl, 4 mM KCl, 1 mM CaCl_2_, 1 mM MgSO_4_, 20 mM HEPES, 1 mM NaH_2_PO_4_, 18 mM glucose, pH 7.4) for 5 min in the absence or presence of Yokuininto. The uptake was terminated by three washes with 0.5 ml of ice-cold Ringer’s solution. Cells were then solubilized in 0.5 ml of 1 N NaOH. After neutralization with 0.5 mL of 1 N HCl, their hydrogen isotype [^3^H] content was assayed by Packard Tri-Carb 2700TR liquid scintillation counter. MDCKII-BCRP transporter assay was determined by the Cihalova method [18]. The Xenopus oocytes system was measured as previously reported [19].

### Drug administration

Male Institute for Cancer Research (ICR) mice (7 weeks) were obtained from Daehan Bio Link company (DBL, Eumseong, Korea). The animals were kept in a clean animal room under specific pathogen free conditions. Mice were allowed to adapt to the environment for a week before being used for the experiment. All animals were housed under 12 h light–12 h dark cycle, lights at 10:00 am. Housing room temperature was maintained at 20–24 °C and humidity at 40–60%. Prior to experimental testing, they were housed in groups of four in standard cages containing a supply of food pellets and water. Mice were assigned as seven per each group to explore the feasibility: (1) control group (*n* = 7; intraperitoneally vehicle injected plus vehicle treated group), (2) PO 400 mg/kg/day group (n = 7; intraperitoneally PO injected plus vehicle treated group), (3) Allopurinol 50 mg/kg/day group (n = 7; intraperitoneally allopurinol injected plus vehicle treated group), and (4) PO + K-25 300 mg/kg/day group (n = 7; intraperitoneally PO plus K-25 treated group). Mice were treated with 400 mg/mL PO for 3 days, and then K-25 was administered intraperitoneally once a day for 3 days. All the procedures were randomly ordered between the groups. At the end of the experiment, mice were sacrificed by CO_2_ inhalation. Animal maintenance and treatment were conducted in compliance with the Principles of Laboratory Animal Care. All animal procedures were approved by the institutional animal care and use committee of Korea Institute of Oriental Medicine (Approval No. KIOM #16–069).

### Immunofluorescence analysis

Cells were fixed through incubation with 4% paraformaldehyde at room temperature for 30 min. Fixed cells were rinsed in PBS, treated with 0.5% BSA for 30 min, and then incubated overnight at 4 °C with the rabbit anti-GLUT9 and anti-OAT3 antibodies. They were then incubated for 2 h with an Alexa 488 Fluor-conjugated secondary antibody. The cells were finally washed in PBS and mounted using Vectashield Mounting Medium containing DAPI. Immunofluorescent images were captured using microscope (Olympus Microscope System BX51; Olympus).

### Xanthine oxidase inhibition (XOI) activity and uric acid assay

XO catalyzes the conversion of hypoxanthine to xanthine and then uric acid as a final product in the presence of molecular oxygen to yield superoxide anion [20]. Inhibitory effects on xanthine oxidase activity were measured by a decrease in uric acid formation. Urine and serum level of uric acid were determined by the uric acid detection kit (ab65344, abcam). The experimental process performed according to manufacturer’s instructions.

### Statistical analysis

The variables including primary ones, serum and urinary uric acid concentrations were expressed as the mean ± standard error of the mean (S.E.M.). The statistical variables were analyzed using a one-way analysis of variance (ANOVA) and post hoc multiple mean comparisons (Bonferroni test). All variables were analyzed using the GraphPad Prism 5.10 software (GraphPad Software Inc., USA).

## Results

### K-25 reduces serum level of uric acid

**Fig. 1:**
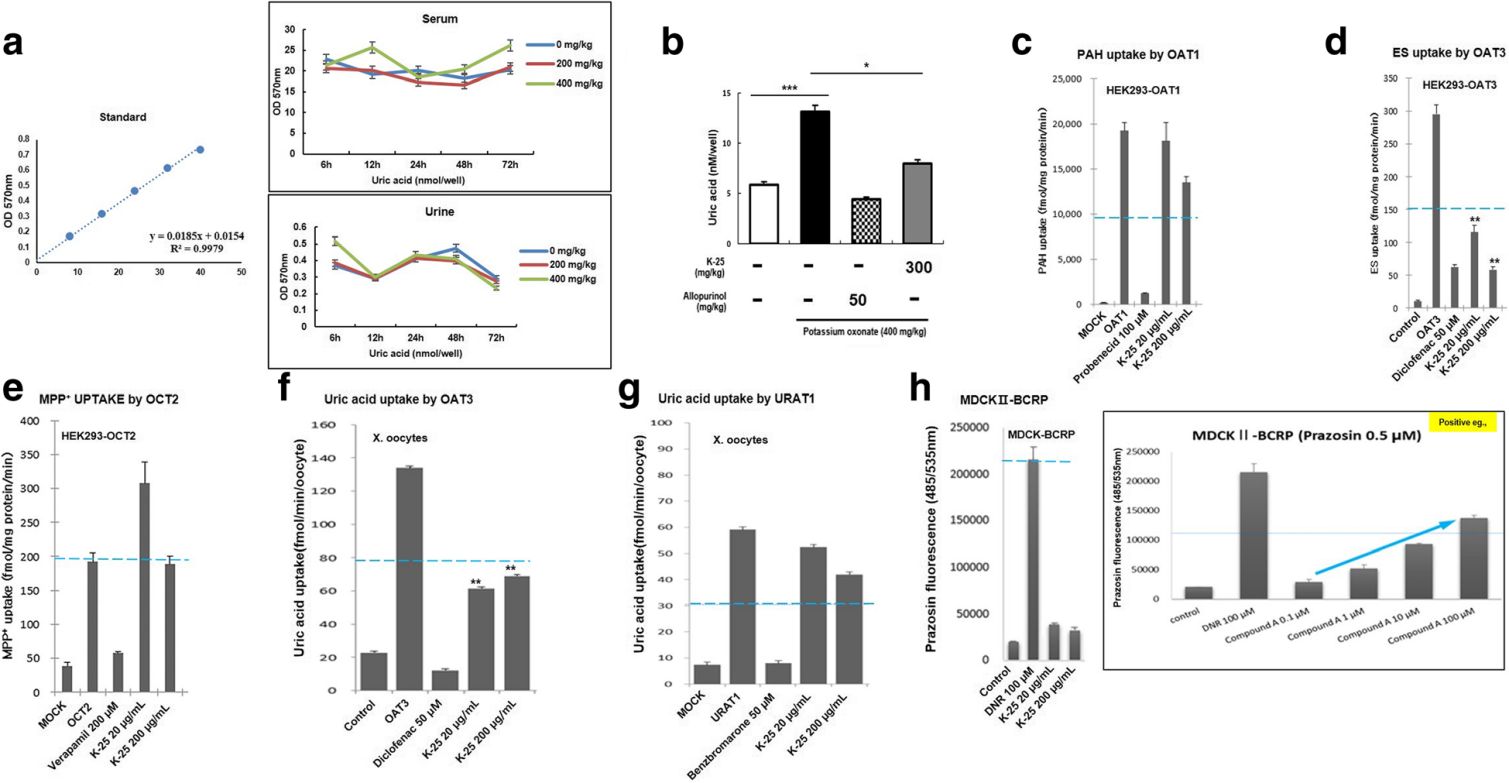
Inhibition of uric acid and transporters by K-25 in vivo model. **a** In vivo uric acid level was determined using the uric acid assay kit. Uric acid detection analyses showed urine and serum levels of uric acid at 6–72 h after PO injection in different groups of mice as indicated. Concentration of PO: 0 mg/mL (blue line), 200 mg/mL (red line), 400 mg/mL (green line). **b** Effects of K-25 on serum in hyperuricemia mice. Mice were pretreated with 400 mg/mL of PO for 3 days prior to 300 mg/mL of K-25 treatment for 72 h. Values are mean ± standard error of the mean. **p < 0.01, compared to the PO-treated control. The specific substrates used were (**c**) [^3^H]PAH for OAT1, (**d**) [^3^H]ES for OAT3, and (**e**) [^3^H]MPP^+^ for OCT2 in the HEK293 overexpressing system. (**f**, **g**) Inhibitory effect of K-25 on uric acid uptake by OAT1 and URAT1 transporters in Xenopus oocytes. **h** Transcellular transport of prototypical substrates of BCRP was measured using monolayers of MDCK cells transfected with the MDCK genes, or of MDCK-WT control cells seeded on transwell membranes. Prazosin (2 μM), a substrate of BCRP was incubated with the MDCK-BCRP cell lines. All values are mean ± SD (triplicate in each experiment; each experiment was repeated three times). **p < 0.01, compared to OAT3. The statistical significance (*p < 0.05, **p < 0.01, ***p < 0.001) was determined using an one-way analysis of variance (ANOVA) with Bonferroni correction

The serum level of uric acid in PO-induced mice increased markedly at 72 h, whereas the urine level decreased at 48 and 72 h. PO (400 mg/kg) markedly increased the serum level of uric acid (Fig. 1a). Compared to that of the untreated mice group, the serum level of uric acid in the PO-induced mice was markedly increased by approximately 44.9% after PO administration. K-25 (300 mg/kg) significantly reduced serum level of uric acid by approximately 44% compared to that of PO-induced mice. The in vivo anti-hyperuricemia efficacy of K-25 was analyzed using a uric acid ELISA in the PO-induced mouse model (Fig. 1b). We evaluated the transport characteristics of K-25 using HEK293 cells with overexpressing uptake for OAT1, OAT3 and OCT2 and also the efflux transport interactions of K-25 with representative inhibitors using Xenopus oocytes for URAT1 and BCRP. The two showed the profiles without any remarkable changes. We also evaluated OAT1, OAT3, OCT2 and URAT1-mediated K-25 transport in the presence of representative inhibitors of those transporters: probenecid, diclofenac, verapamil and benzbromarone, respectively. Only diclofenac inhibited the OAT3-mediated K-25 transport. In contrast, K-25 did not markedly inhibit the activities of OAT1, OCT2, URAT1, and BCRP in the concentration ranges tested (Fig. 1c-h). Those results suggest that K-25 is actively taken up into cells via the OAT3 transporter.

### K-25 inhibits NO synthesis and IL-1α secretion by LPS

**Fig. 2:**
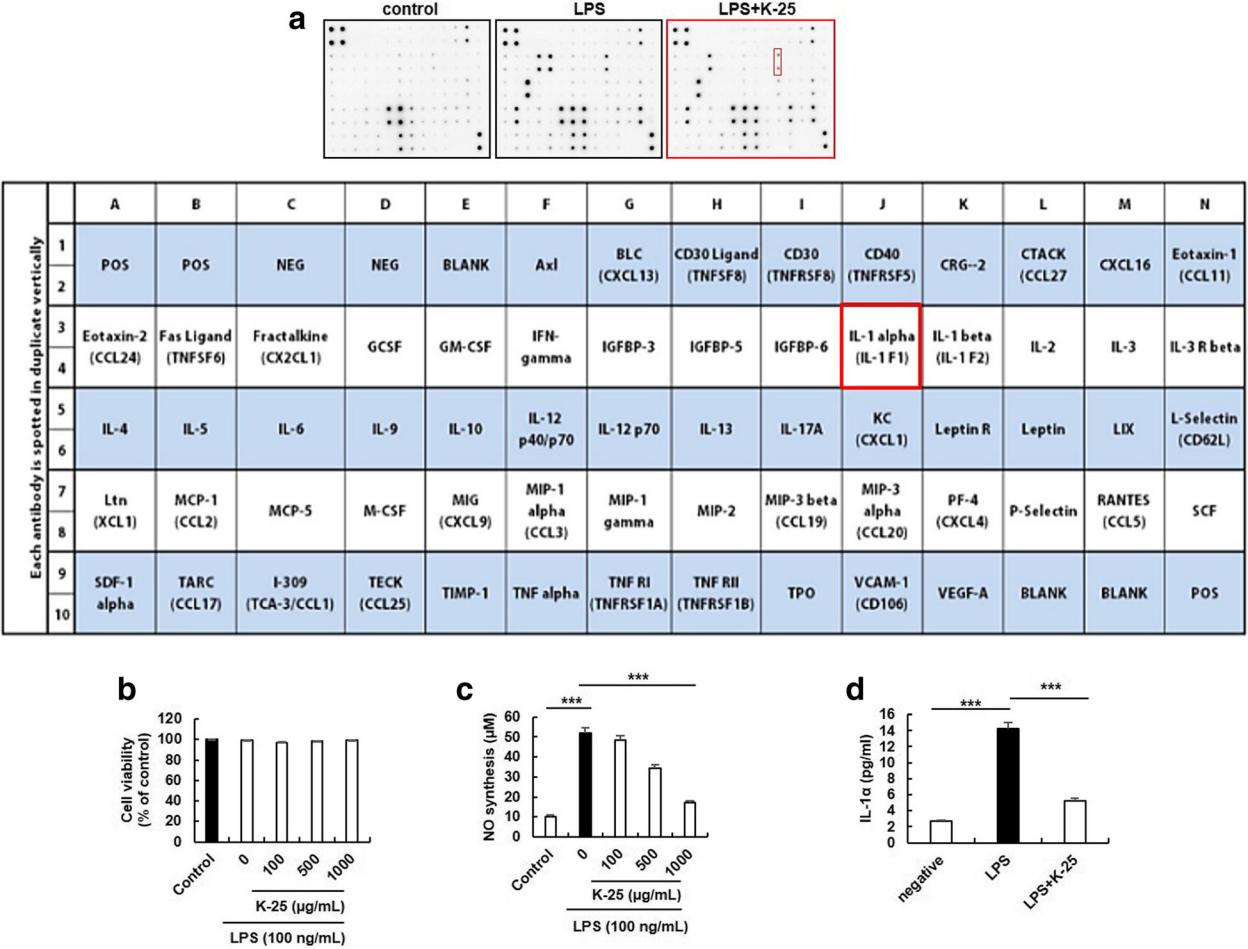
Inhibition of LPS-induced NO production and IL-1α secretion by K-25. Raw 264.7 cells were pretreated with various concentrations of K-25 for 1 h before LPS (100 ng/mL) treatment. **a** After 24 h-incubation, the cultures were subjected to cytokine antibody array assay. **b** After 24 h-incubation, cell viability was measured using the EZ-cytox assay kit. **c** After 24 h of incubation, NO production was measured using the Griess Reagent System assay. **d** After 24 h-incubation, the culture medium was assayed using ELISA for IL-1α. Values represent mean ± standard error of the mean. **p < 0.01, compared to the LPS control. The statistical significance (*p < 0.05, **p < 0.01, ***p < 0.001) was determined using ANOVA with Bonferroni correction

To test the effects of K-25 on the generation of inflammatory cytokines, the amounts of G-CSF, GM-CSF, IL-6, IL-1α, MIP-2, CCL5, and TNFR2 in the LPS-induced macrophages were measured using a cytokine antibody array (Fig. 2a). In particular, increased IL-1α was suppressed by Yokuininto. Macrophage cells were treated with various concentrations of LPS (0, 50, 100, 500 ng/mL) for 24 h and cell viability was measured. LPS showed no cytotoxic effect on the macrophage cells (Fig. 2b and Additional file 1: Figure S1). We assessed the non-cytotoxic concentrations of LPS in Raw264.7 cells using MTT assays. As shown in Additional file 1: Figure S1, cell viability was not significantly changed after the LPS treatment up to 100 ng/ml for 24 h compared with the untreated control cells. Thus, we used LPS at 100 ng/ml concentration in all subsequent experiments. LPS (100 ng/mL) treatment strongly induces the synthesis of NO involved in inflammation (Fig. 2c). In addition, the 500 and 1000 μg/mL K-25 inhibited NO synthesis by approximately 33.7 and 64.6%, respectively, compared to that of LPS-treated macrophages (Fig. 2c). K-25 significantly inhibited the LPS-induced secretion of IL-1α by approximately 63.2% compared to the LPS-treated macrophages (Fig. 2d). Those results suggest that LPS-induced NO synthesis and IL-1α secretion are inhibited by K-25.

### OAT3 and GLUT9 transporter levels are altered by PO-induced hyperuricemic condition

**Fig. 3:**
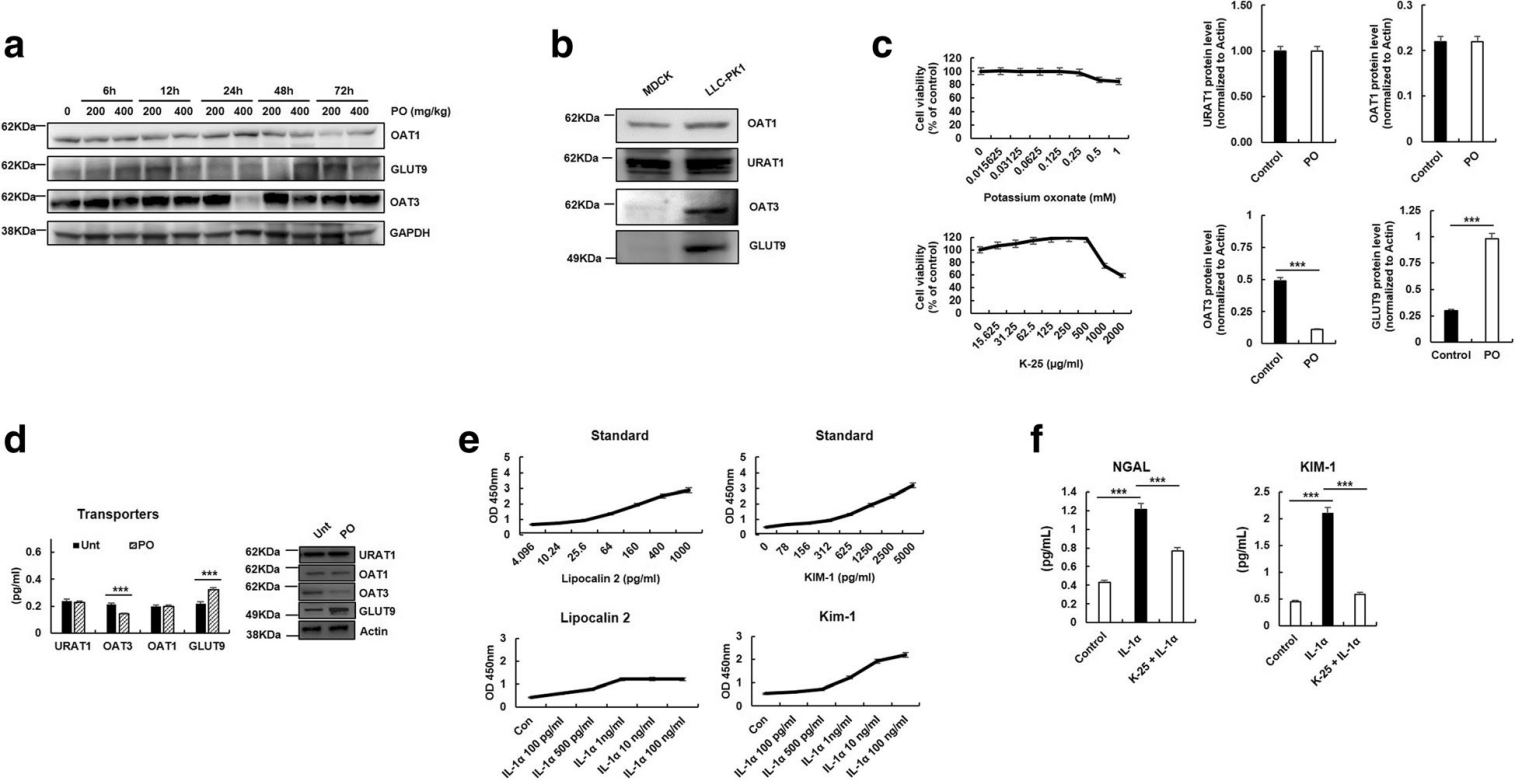
Analysis of transporters and inflammatory factors. **a** Protein levels of transporters OAT1, OAT3 and URAT1 were determined using immunoblot analysis in kidney cell lines. **b** After treatment of LLC-PK1 cells with PO and K-25 for 24 h, cell viability was measured using the cell viability assay. **c** Protein levels of transporters OAT1, OAT3, GLUT9 and URAT1 were determined using ELISA and immunoblot analysis in PO-induced LLC-PK1 cells. **d** Activation of NGAL and KIM-1 by IL-1α. The protein levels in IL-1α-treated LLC-PK1 cells were detected using ELISA. **e** LLC-PK1 cells were stimulated with IL-1α (10 ng/mL) with or without pretreatment with K-25 (0.5 mg/mL). **f** After 24 h-incubation, NGAL and KIM-1 levels were measured using ELISA. Values represent mean ± standard error of the mean. **p < 0.01, compared to the IL-1α control. The statistical significance (*p < 0.05, **p < 0.01, ***p < 0.001) was determined using ANOVA with Bonferroni correction

Hyperuricemia is closely involved in the regulation of renal transporters, including OAT1, OAT3, GLUT9 and URAT1. Thus, we examined the change of the expression of renal transporters under hyperuricemic condition using ELISA and immunoblot analysis in renal proximal epithelial cell lines. Especially, in LLC-PK1 cells, OAT3 levels decreased significantly in PO-induced hyperuricemic condition, whereas the GLUT9 transporter levels were markedly increased. However, PO did not alter the levels of URAT1 (Fig. 3a-d). IL-1α, a pro-inflammatory cytokine, markedly elevated NGAL and KIM-1 activities in LLC-PK1 cells (Fig. 3e). K-25 significantly inhibited NGAL and KIM-1 activities of IL-1α-treated LLC-PK1 cells by approximately 36.9 and 72.1%, compared to those of the IL-1α treated group (Fig. 3f). Those results suggest that PO induces the change in OAT3 and GLUT9 transporters. In addition, NGAL and KIM-1 activated by IL-1α are inhibited by K-25.

### K-25 improves hyperuricemia via OAT3, GLUT9, and XO

**Fig. 4:**
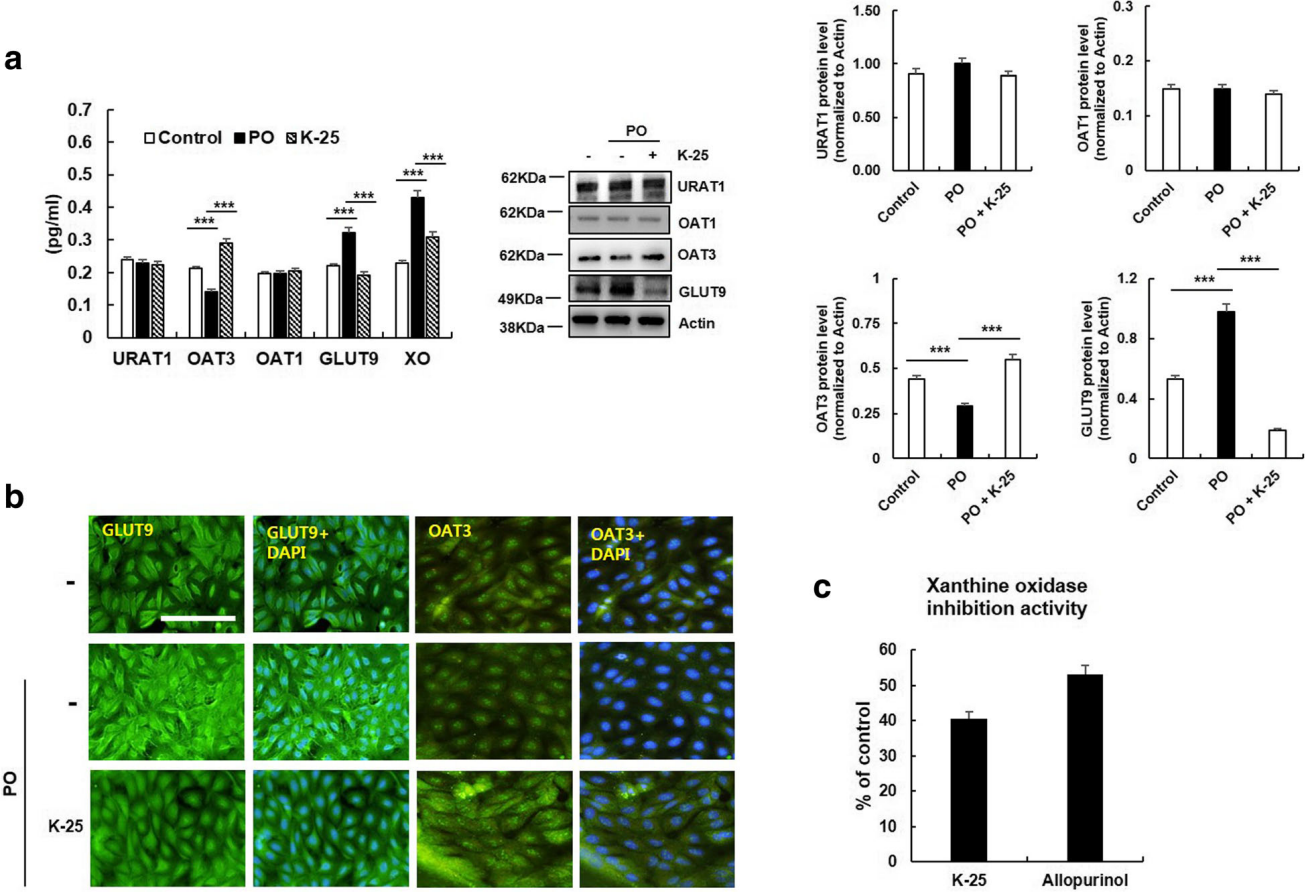
Inhibition of PO-induced hyperuricemia by K-25. LLC-PK1 cells were stimulated with PO (0.25 mM) with or without pretreatment with K-25 (0.5 mg/mL). **a** After 24 h of incubation, OAT1, OAT3, URAT1, GLUT9, and XO were measured using an immunoblot assay. **b** LLC-PK1 cells were incubated with K-25 for 24 h, and the intracellular levels of OAT3 and GLUT9 transporters were analyzed using immunofluorescence analysis. Scale bar = 50 μm. **c** Effect of K-25 on XO inhibition activity. Values are represented as means ± SEM, *p < 0.05, versus the allopurinol group. The statistical significance (*p < 0.05, **p < 0.01, ***p < 0.001) was determined using ANOVA with Bonferroni correction

The effect of K-25 on the levels of OAT3 and GLUT9 in hyperuricemic condition is shown in Fig. 4a and b. K-25 treatment significantly downregulated GLUT9, but upregulated OAT3 in PO-induced LLC-PK1 cells. In addition, K-25 significantly decreased the XO activity in LLC-PK1 cells under hyperuricemic condition (Fig. 4c). Those results suggest that K-25 acts not only as an OAT3 and GLUT9 transporters but also functions as a XOI.

## Discussion

In this study, we observed that Yokuininto decreased the urine level of uric acid in PO-induced hyperuricemic mice. Subsequently, we demonstrated that Yokuininto inhibited the XO activity in renal proximal epithelial cells and regulated the expression of OAT3 and GLUT9, which are important renal uric acid transporters. Furthermore, Yokuininto suppressed purinergic pathway-related inflammation and protected against kidney dysfunction via NGAL and KIM-1 under hyperuricemic condition.

The progress of pathological conditions such as gout is a metabolic illness associated with hyperuricemia. The relationship between pathogenesis of gout and hyperuricemia has been previously reported [21]. Allopurinol is effective in treating chronic symptoms but is associated with adverse side effects such as fever, rash or liver and kidney disorders [22]. Unfortunately, uricosuric agents used for clinical applications of hyperuricemia and gout are scarce [23], and investigations for identifying novel anti-hyperuricemia agents have attracted much attention. Several animal models of human hyperuricemia have been used to select novel hyperuricemia agents. PO is an extensively-used experimental reagent that triggers hyperuricemia in rodents because PO can inhibit uricase, the enzyme that degrades uric acid to allantoin [24]. Hence, we used the PO-induced hyperuricemia mice in the study. After three days of PO administration, serum level of uric acid in PO-induced mice markedly increased by approximately 44.9% compared to the levels in untreated mice. Interestingly, Yokuininto administration significantly reduced serum level of uric acid in PO-induced hyperuricemic mice. It suggests that Yokuininto exerts uricosuric effect in hyperuricemic mice.

Uric acid excretion and reabsorption in kidneys relies on the function of uric acid transporters. Studies show that a variety of uric acid transporters modulate uric acid [25]. In patients with gout, the inability of kidneys to absorb and excrete uric acid is the dominant cause of hyperuricemia [26]. The efflux mechanism for hydrophilic organic anions is regulated by OAT3. A gene knockout study in mice indicated that the absence of OAT3 decreases uricosuria, suggesting that its principal function is uric acid excretion [27]. Another key regulator of uric acid absorption is GLUT9, which is highly expressed on the surface of renal proximal tubular cells [28]. A genetic study showed that GLUT9 is the major transporter associated with high plasma uric acid levels [29]. In addition to their association with uric acid levels, a significant connection of GLUT9 and OAT3 with gout was reported [30]. In this study, PO administration markedly decreased OAT3 levels and increased GLUT9 levels. Those results may demonstrate that PO might reduce uric acid excretion and induce uric acid reabsorption. Yokuininto significantly increased OAT3 levels and decreased GLUT9 levels, which suggested that Yokuininto might enhance uric acid excretion and suppress uric acid reabsorption. Taken together, our results suggested that Yokuininto increased the urine level of uric acid via increasing uric acid excretion and decreasing renal uric acid production through the reabsorption pathways.

Compared to the frequency of occurrence of genetic syndromes related to IL-1-regulated illness, crystal-induced arthropathy is highly prevalent. IL-1 activation and release triggers a massive inflammatory response, with rapid recruitment of immune cells to the region of crystal deposition, resulting in an acute event of gout. In this study, LPS treatment significantly increased IL-1α release as well as NO production in macrophages. Yokuininto significantly and dose-dependently inhibited NO production, as well as IL-1α secretion. Those results suggested that Yokuininto might inhibit uric acid production via the inhibition of both pro-inflammatory response and reactive oxygen species generation because XO catalyzes the reduction of O_2_ to H_2_O_2_ and superoxide.

Accumulation of uric acid can damage kidney cortex and other organells by inducing inflammatory cytokines. Therefore, we examined the effects of Yokuininto on the expression of pro-inflammatory cytokines IL-1α; acute kidney injury molecules: NGAL and KIM-1. [7]. They are crucial factors in the development of renal inflammatory response [31]. Those results suggest that Yokuininto may suppress inflammation and protect against kidney dysfunction in hyperuricemia. The present findings demonstrated that Yokuininto lowered serum level of uric acid through both increased uric acid excretion and decreased uric acid production. Moreover, Yokuininto markedly increased XOI activity and anti-inflammatory activities.

## Conclusions

In conclusion, our data demonstrated that Yokuininto reduced uric acid in serum of PO-induced hyperuricemic mice through two pathways. First, in hyperuricemic condition, Yokuininto markedly increased OAT3 expression, as well as decreased GLUT9 expression, and enhanced urate secretion and inhibited urate reabsorption. Second, Yokuininto significantly increased XOI activity, and thus might directly inhibit uric acid production by inhibiting the catalytic activity of XO. Furthermore, we identified Yokuininto, the inflammatory cytokine inhibitor. Yokuininto inhibited NGAL, KIM-1 and IL-1α.

## Additional file

Additional file 1: **Figure S1.** Effect of LPS on Raw 264.7 cell viability. Cells were treated with 0–1000 ng/ml LPS for 24 h. The viability of the cells was measured by the MTT assay. The statistical significance (*p < 0.05, **p < 0.01, **p < 0.001) was determined using ANOVA with Bonferroni correction (JPG 44 kb)

## Abbreviations

GLUT9: glucose transporter 9
IL-1α: Interleukin-1 alpha
KIM-1: kidney injury molecule-1
LPS: Lipopolysaccharide
NGAL: neutrophil gelatinase-associated lipocalin
OAT3: organic anion transporter 3
PO: potassium oxonate
sUA: serum uric acid
XO: xanthine oxidase

## References

[1] Chen CJ, Kono H, Golenbock D, Reed G, Akira S, Rock KL (2007). Identification of a key pathway required for the sterile inflammatory response triggered by dying cells. Nat Med.

[2] Feng J, Li X, Yang X, Zhang C, Yuan Y, Pu J (2010). A new practical system for evaluating the pharmacological properties of uricase as a potential drug for hyperuricemia. Arch Pharm Res.

[3] Amat N, Umar A, Hoxur P, Anaydulla M, Imam G, Aziz R (2015). Traditional Uighur Medicine Karapxa decoction, inhibits liver xanthine oxidase and reduces serum uric acid concentrations in hyperuricemic mice and scavenges free radicals in vitro. BMC Complement Altern Med.

[4] Lee SH, Park G, Kim SB, Oh DS (2018). Uric acid-lowering effect and intestinal permeability of Kampo medicine, Hachimijiogan, Yokuininto and Goshakusan. Eur J Intern Med.

[5] Ammon HP (2010). Modulation of the immune system by Boswellia serrata extracts and boswellic acids. Phytomedicine..

[6] Pimentel SP, Barrella GE, Casarin RC, Cirano FR, Casati MZ, Foglio MA (2012). Protective effect of topical Cordia verbenacea in a rat periodontitis model: immune-inflammatory, antibacterial and morphometric assays. BMC Complement Altern Med.

[7] Oh DR, Kim JR, Choi CY, Choi CH, Na CS, Kang BY, et al. Effects of ChondroT on potassium Oxonate-induced Hyperuricemic mice: downregulation of xanthine oxidase and urate transporter 1. BMC Complement Altern Med. 2019;19:10. 10.1186/s12906-018-2415-2. 10.1186/s12906-018-2415-2 PMC6323677 30621705

[8] Bellomo R, Kellum JA, Ronco C (2012). Acute kidney injury. Lancet.

[9] Oh SM, Park G, Lee SH, Seo CS, Shin HK, Oh DS (2017). Assessing the recovery from prerenal and renal acute kidney injury after treatment with single herbal medicine via activity of the biomarkers HMGB1, NGAL and KIM-1 in kidney proximal tubular cells treated by cisplatin with different doses and exposure times. BMC Complement Altern Med.

[10] Wang YH, Liu YH, He GR, Lv Y, Du GH (2015). Esculin improves dyslipidemia, inflammation and renal damage in streptozotocin-induced diabetic rats. BMC Complement Altern Med.

[11] Yang L, Brooks CR, Xiao S, Sabbisetti V, Yeung MY, Hsiao LL (2015). KIM-1–mediated phagocytosis reduces acute injury to the kidney. J Clin Invest.

[12] Kuehn EW, Hirt MN, John AK, Muehlenhardt P, Boehlke C, Pütz M (2007). Kidney injury molecule 1(Kim1) is a novel ciliary molecule and interactor of polycystin 2. Biochem Biophys Res Commun.

[13] Kosuke T, Tadahisd U, Toshikazu Y, Toru Y, Tadasho H, Kenro K. [Yokuininto (kampo medicine): a choice for mild to moderate osteoarthritis of the knee joint]. Kampo and the newest therapy 2007–02;16:61–66. (In Janpanese). http://ci.nii.ac.jp/naid/40015368083/.

[14] Toshiaki K, Atsushi N, Hiroshi F, Takahiro S, Yutaka S, Katsutoshi T. [The Consideration of the Patients with Rheumatoid Arthritis Successfully Treated with Yokuinin-to-kami]. Journal of the Japan Society for Oriental Medicine 2001;51:51–59.

[15] Kracie Holdings,Ltd [http://www.kracie.co.jp/products/ph/1202036_2220.html]. Accessed 7 Feb 2018.

[16] Rakuten, Inc Rakuten Global Market [https://global.rakuten.com/en/store/yoikenkou/item/103-4987045046704_1/]. Accessed 5 Mar 2019.

[17] Kimura N, Masuda S, Tanihara Y, Ueo H, Okuda M, Katsura T (2005). Metformin is a superior substrate for renal organic cation transporter OCT2 rather than hepatic OCT1. Drug Metab Pharmacokinet.

[18] Cihalova D, Ceckova M, Kucera R, Klimes J, Staud F (2015). Dinaciclib, a cyclin-dependent kinase inhibitor, is a substrate of human ABCB1 and ABCG2 and an inhibitor of human ABCC1 in vitro. Biochem Pharmacol.

[19] Katakura M, Kudo N, Tsuda T, Hibino Y, Mitsumoto A, Kawashima Y (2007). Rat organic anion transporter 3 and organic anion transporting polypeptide 1 mediate perfluorooctanoic acid transport. Int J Health Sci Res.

[20] McCord JM, Fridovich I (1968). The reduction of cytochrome c by milk xanthine oxidase. J Biol Chem.

[21] Neogi T (2011). Gout. N Engl J Med.

[22] Haidari F, Jr M, Shahi M, Keshavarz SA, Rashidi MR (2009). Inhibitory Effects of Tart Cherry (Prunus cerasus) Juice on Xanthine Oxidoreductase Activity and its Hypouricemic and Antioxidant Effects on Rats. Malays J Nutr.

[23] Pillinger MH, Keenan RT (2008). Update on the management of hyperuricemia and gout. Bull NYU Hosp Jt Dis.

[24] Wu XH, Yu CH, Zhang CF, Anderson S, Zhang YW (2014). Smilax riparia reduces hyperuricemia in mice as a potential treatment of gout. Am J Chin Med.

[25] So A, Thorens B (2010). Uric acid transport and disease. J Clin Invest.

[26] Perez-Ruiz F, Calabozo M, Erauskin GG, Ruibal A, Herrero-Beites AM (2002). Renal underexcretion of uric acid is present in patients with apparent high urinary uric acid output. Arthritis Rheum.

[27] Eraly SA, Vallon V, Rieg T, Gangoiti JA, Wikoff WR, Siuzdak G (2008). Multiple organic anion transporters contribute to net renal excretion of uric acid. Physiol Genomics.

[28] Wu XH, Zhang J, Wang SQ, Yang VC, Anderson S, Zhang YW (2014). Riparoside B and timosaponin J, two steroidal glycosides from Smilax riparia, resist to hyperuricemia based on URAT1 in hyperuricemic mice. Phytomedicine.

[29] Li S, Sanna S, Maschio A, Busonero F, Usala G, Mulas A (2007). The GLUT9 gene is associated with serum uric acid levels in Sardinia and Chianti cohorts. PLoS Genet.

[30] Döring A, Gieger C, Mehta D, Gohlke H, Prokisch H, Coassin S (2008). SLC2A9 influences uric acid concentrations with pronounced sex-specific effects. Nat Gene.

[31] Nguyen TD, Thuong PT, Hwang IH, Hoang TK, Nguyen MK, Nguyen HA (2017). Anti-Hyperuricemic, Anti-Inflammatory and Analgesic Effects of Siegesbeckia orientalis L. Resulting from the Fraction with High Phenolic Content. BMC Complement Altern Med.

