# Supplementary figures and images for: Dual actions on gout flare and acute kidney injury along with enhanced renal transporter activities by Yokuininto, a Kampo medicine
Source: BMC Complement Altern Med. 2019 Mar 12;19:57. doi: 10.1186/s12906-019-2469-9 (PMC6419507; doi:10.1186/s12906-019-2469-9)

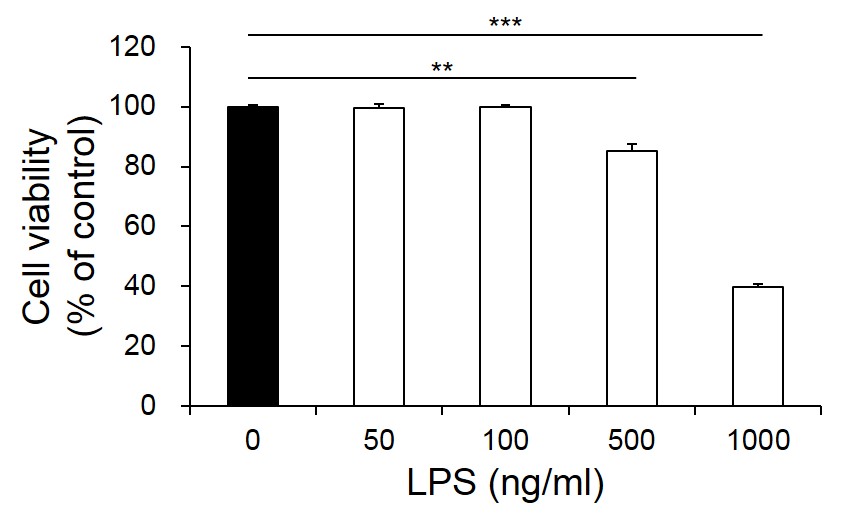

Supplement: Supplementary file 1 — Figure S1. Effect of LPS on Raw 264.7 cell viability. Cells were treated with 0–1000 ng/ml LPS for 24 h. The viability of the cells was measured by the MTT assay. The statistical significance (*p < 0.05, **p < 0.01, **p < 0.001) was determined using ANOVA with Bonferroni correction (JPG 44 kb) [file 12906_2019_2469_MOESM1_ESM.jpg]
